# Breast carcinoma: atypical presentation

**DOI:** 10.11604/pamj.2025.52.148.46386

**Published:** 2025-12-08

**Authors:** Rajiv Sonarkar, Sanchi Sonarkar

**Affiliations:** 1 Department of Surgery, Datta Meghe Medical College, Nagpur, Datta Meghe Institute of Higher Education and Research (Deemed University), Sawangi, Wardha, India

**Keywords:** Breast carcinoma, wart, cutaneous melanoma

## Image in medicine

A 60-year-old lady presented with a solitary wart-like lesion on the left breast, with no associated pain, erythema, or discharge, initially suggesting a benign dermatological condition. Breast carcinoma with cutaneous involvement occurs in 20-30% of advanced cases, and infiltrating ductal carcinoma is the most common subtype (80-85%). However, wart-like cutaneous manifestations are exceedingly rare. Clinical examination of the lesion was firm, irregular, and palpation of the breast revealed an underlying, indistinct firmness, and imaging (mammography, ultrasound) was inconclusive, so a biopsy was done for confirmation. Histopathology showed tumour cells infiltrating the dermis with keratinisation or verrucous overgrowth, establishing ductal carcinoma of the breast with an atypical cutaneous presentation. Such lesions, rarely reported in the literature, may result from tumour spread via lymphatics or dermal invasion and often mimic benign conditions such as papilloma, dermatofibroma, or infections, leading to delayed diagnosis. Therapeutic intervention included surgery (modified radical mastectomy) with further management planned based on receptor status and standard oncologic protocols. Short-term recovery was uneventful. Medium-term management and prognosis are being guided by the final tumour stage and receptor profile, with plans for regular follow-up.

**Figure 1:**
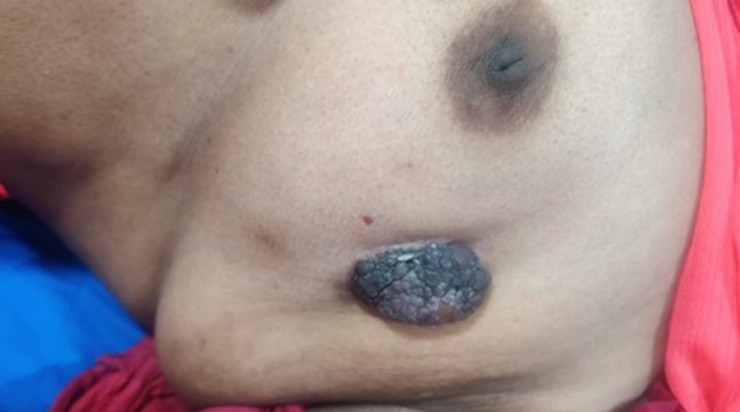
atypical presentation of breast cancer

## Available online at

https://www.panafrican-med-journal.com//content/article/52/148/full

